# Scientific integrity 2.0: misconduct. Let's prevent, not punish!

**DOI:** 10.31744/einstein_journal/2019ED5064

**Published:** 2019-06-25

**Affiliations:** 1 Instituto Israelita de Ensino e Pesquisa Albert Einstein, Hospital Israelita Albert Einstein, São Paulo, SP, Brazil; 2 Hospital Israelita Albert Einstein, São Paulo, SP, Brazil

The growing complexity of modern, innovative research has brought up important issues concerning research compliance and responsible conduct of research. The scope of items to be managed have expanded to include a great variety of issues, ranging from authorship, plagiarism, data management, confidentiality, patent rights, conflicts of interest to ethical conduct, animal well-being and social aspects of ongoing research.^(^ ^1^ ^)^ All these factors have increasingly burdened the daily routine of wet laboratories, clinical research centers, and animal facilities involved in scientific endeavor.

A clear effect resulting from the worldwide boom in science and innovation is the increase in retractions of papers by authors, journals, and industry partners. Retractions are in the spotlight and have gained even more notoriety after the launching of the Retraction Watch Database (http://retractiondatabase.org/RetractionSearch.aspx?) on October 25, 2018.^(^ ^2^ ^)^ This state of affairs has to be dealt with by the scientific community, academic institutions, compliance managers, and stakeholders in general. This is partly driven by the perception of the piling up of retractions − approximately 500 per year. As a result, there is a mounting interest that academic institutions take a more proactive role in helping researchers, graduate students, and collaborating partners to comply with research integrity. The first step to ensure great rigor in performing research is to comply with organizational rules and norms, which can be done with assistance by research management.^(^ ^3^ ^)^

At the *Hospital Israelita Albert Einstein* (HIAE), in São Paulo (SP, Brazil), active research management has been established since 2014, and proven of great value to improve scientific development throughout the organization. A step further was taken when the Research Integrity Office was implemented at HIAE, in June 2017. In a pioneering effort to enhance research quality, the office carries out preemptive auditing of ongoing research and of recently published articles to help ensure research integrity and responsible conduct of research. This issue has been very recently further highlighted, since international well-known universities acknowledged the need for a more decisive approach to upgrade research reproducibility and responsible research practices.

Mayer et al.,^(^ ^4^ ^)^ recently pointed out, in their editorial “Step up for quality research”, and Schrag et al.,^(^ ^5^ ^)^ rightly recommended greater involvement of universities to ensure their own research communities produce high-quality, reproducible research data.^(^ ^5^ ^)^ They also reported that to introduce greater rigor into the study of research integrity and into the factors fostering or discouraging responsible behavior, the participants at the Fifth World Conference on Research Integrity endorsed the “Amsterdam Agenda”.^(^ ^6^ ^)^ This document officially describes plans to establish a registry of investigations on research integrity related topics and to foster good practices and research in this area.

With the continuous work done by the Research Integrity Office at HIAE, we hope to promote a fruitful communication channel with all those involved in biomedical and clinical research and healthcare management, aiming to benefit the health and wellbeing of patients and society alike.
